# Oxytocin Increases Phasic and Tonic GABAergic Transmission in CA1 Region of Mouse Hippocampus

**DOI:** 10.3389/fncel.2019.00178

**Published:** 2019-05-07

**Authors:** Claudia Maniezzi, Francesca Talpo, Paolo Spaiardi, Mauro Toselli, Gerardo Biella

**Affiliations:** ^1^Laboratory of Physiology, Department of Biology and Biotechnology “Lazzaro Spallanzani”, University of Pavia, Pavia, Italy; ^2^Neurophysiology Unit, Department of Brain and Behavioral Sciences, University of Pavia, Pavia, Italy

**Keywords:** oxytocin, GABA, hippocampus, patch-clamp, IPSC

## Abstract

Oxytocin is a neuropeptide that plays important peripheral and central neuromodulatory functions. Our data show that, following activation of oxytocin receptors (OtRs) with the selective agonist TGOT (Thr^4^,Gly^7^-oxytocin), a significant increase in frequency and amplitude of spontaneous inhibitory postsynaptic currents (sIPSC) occurred in hippocampal CA1 pyramidal neurons (PYR) in mice. TGOT affected also sIPSC deactivation kinetics, suggesting the involvement of perisynaptic GABA_A_ receptors (GABA_A_Rs) as well. By contrast, TGOT did not cause significant changes in frequency, amplitude or deactivation kinetics of miniature IPSC, suggesting that the effects elicited by the agonist are strictly dependent on the firing activity of presynaptic neurons. Moreover, TGOT was able to modulate tonic GABAergic current mediated by extrasynaptic GABA_A_Rs expressed by PYRs. Consistently, at spike threshold TGOT induced in most PYRs a significant membrane hyperpolarization and a decrease in firing rate. The source of increased inhibition onto PYRs was represented by stuttering fast-spiking GABAergic interneurons (INs) that directly respond to TGOT with a depolarization and an increase in their firing rate. One putative ionic mechanism underlying this effect could be represented by OtR activation-induced up-modulation of L-type Ca^2+^ channels. In conclusion, our results indicate that oxytocin can influence the activity of a subclass of hippocampal GABAergic INs and therefore regulate the operational modes of the downstream PYRs by increasing phasic and tonic GABAergic transmission in CA1 region of mouse hippocampus.

## Introduction

Oxytocin (OT) is a small neuropeptide that plays important peripheral (i.e., parturition and lactation) and central neuromodulatory functions. OT is mostly synthesized in the magnocellular neurons of the supraoptic nuclei and paraventricular nuclei of the hypothalamus, projecting to the posterior pituitary gland, where it is released into the bloodstream to exert its peripheral effects. OT is also synthesized in the parvocellular neurons of the hypothalamus projecting to several distinct brain regions (Ludwig and Pittman, 2003) where it operates as a neuromodulator (Raggenbass, 2001). Within the central nervous system OT is involved in social recognition, social exploration, as well as anxiety and fear-related behavior (Donaldson and Young, 2008; Heinrichs et al., 2009). A link between autism spectrum disorders and the OT system has been proposed as well (Francis et al., 2016; Guastella and Hickie, 2016).

Among the brain areas in which receptors for OT (OtR) have been widely demonstrated there is the CA1 field of the hippocampal formation (Tribollet et al., 1988; Yoshimura et al., 1993; Barberis and Tribollet, 1996; Campbell et al., 2009; Ophir et al., 2012). Several electrophysiological studies have shown that OT is able to exert direct effects on specific neuronal populations in the CA1 area of the rat (Mühlethaler et al., 1983; Mühlethaler et al., 1984; Raggenbass et al., 1989; Raggenbass, 2001). In particular, OT seems to exert an excitatory action on a specific class of CA1 GABAergic interneurons (INs) and in turn to enhance inhibitory synaptic transmission onto CA1 pyramidal neurons (PYR) in rats (Zaninetti and Raggenbass, 2000; Owen et al., 2013).

Previous works only focused on the role of OT in modulating GABAergic synaptic transmission. At the synapses, GABA exerts its action by activating transiently the GABA_A_ receptors (GABA_A_Rs) expressed on the postsynaptic membrane of PYRs and then giving rise to inhibitory currents responsible for the so-called ‘phasic’ (or synaptic) inhibition (Farrant and Nusser, 2005). However, GABA can escape from the synaptic cleft by spillover and activate GABA_A_Rs located in perisynaptic (Wei et al., 2003) or extrasynaptic (Nusser et al., 1998) sites. In particular, the activation of extrasynaptic receptors gives rise to a persistent (or ‘tonic’) inhibition and several studies reported tonic inhibition in CA1 region of the hippocampus (Banks and Pearce, 2000; Scimemi et al., 2005; Mortensen and Smart, 2006; Pavlov et al., 2009). Then, we hypothesized that OT could also be involved in the modulation of tonic GABAergic transmission.

Taking the cue from those evidences, we tried to confirm, consolidate, and extend the results reported in previous works by characterizing in detail the mechanisms by which OT modulates the neuronal network in the hippocampal CA1 field of the mouse. To this end, we used three groups of animals: (i) wild-type, normally expressing OtRs (Otr^+/+^) mice, (ii) knock-out mice for OtR (Otr^-/-^), and (iii) GAD67-GFP^+^ mice (Δneo) in which GABAergic INs are labeled with Green Fluorescent Protein. First, we evaluated the effect of the selective OtR agonist Thr^4^,Gly^7^-oxytocin (TGOT) on the inhibitory and excitatory synaptic transmission. Then, we tested the hypothesis of the activation of extrasynaptic receptors following GABA spillover, due to an increase in the neurotransmitter release during TGOT perfusion. We also tried to identify the neuronal target of TGOT and to understand the putative ionic mechanism involved in the cellular responses to the agonist. Finally, we elucidated how TGOT influenced the excitability of PYRs and therefore their capability to generate action potentials in response to plasma membrane depolarization.

## Materials and Methods

### Ethics Statement

All animal care and experimental procedures were conducted in accordance with the Italian Animals Scientific Procedures Act. The study protocol was reviewed and approved by the ethical committee of the University of Pavia (OPBA) and by the Italian Ministry for University and Research (Protocol 523/2018 PR) following the ethical policy under which Frontiers in Cellular neuroscience operates. Animals were housed with food and water *ad libitum*, under a 12:12 h light/dark cycle.

### Animals and Brain Slices Preparation

Experiments were performed on young (P17-P26) male and female C57BL/6J Otr^+/+^ mice (obtained from Charles River Laboratories), Otr^-/-^ mice, kindly provided by B. Chini [Institute of Neuroscience, CNR Milano (Lee et al., 2008)], and heterozygous GAD67-GFP^+^ knock-in mice (Tamamaki et al., 2003), kindly provided by Y. Yanagawa (University of Kyoto). Animals were anesthetized by inhalation of isoflurane and decapitated. The head was rapidly submerged in ice-cold (∼4°C), oxygenated (95% O_2_ – 5% CO_2_) cutting solution containing (in mM): Sucrose 70; NaCl 80; KCl 2.5; NaHCO_3_ 26; glucose 15; MgCl_2_ 7; CaCl_2_ 1; NaH_2_PO_4_ 1.25. Transversal 350-μm-thick slices containing the hippocampus were prepared as described by Stoop and Pralong (2000) using a vibratome (DTK-1000, Dosaka EM). Slices were then transferred to an incubation chamber filled with oxygenated artificial cerebrospinal fluid (aCSF) at 37°C, containing (in mM): NaCl 125; KCl 2.5; NaHCO_3_ 26; glucose 15; MgCl_2_ 1.3; CaCl_2_ 2.3; NaH_2_PO_4_ 1.25. After 30 min, brain slices were allowed to equilibrate at room temperature (∼23°C) before electrophysiological analysis.

### Patch-Clamp Recordings

Electrophysiological recordings were performed at room temperature (∼23°C) on submerged slices perfused with oxygenated aCSF at a rate of 0.8 to 1.4 ml/min. The recording chamber was mounted on an E600FN microscope (Nikon) equipped with 4× and 40× water-immersion objectives and connected to a near-infrared CCD camera to allow cells visualization. Records were derived from hippocampal CA1 pyramidal neurons and GABAergic INs located in the *stratum pyramidale*. The former were visually recognized by the triangular shape of their soma in Otr^+/+^ and Otr^-/-^ mice, whereas the latter were identified by their fluorescence in GAD67-GFP^+^ mice. Patch pipettes were prepared from borosilicate glass capillary tubes (Hilgenberg GmbH, Malsfeld, Germany) using a horizontal puller (P-97, Sutter Instruments, Novato, CA, United States) and filled with an intracellular solution iso-osmotic with cytosol. For current clamp experiments and voltage-clamp EPSCs recordings, the following intracellular solution (A) was used: K-gluconate 130 mM; NaCl 4 mM; MgCl_2_ 2 mM; EGTA 1 mM; Hepes 10 mM; creatine phosphate (CP) 5 mM; Na_2_ATP 2 mM; Na_3_GTP 0.3 mM (pH adjusted to 7.3 with KOH). For voltage-clamp IPSC recordings, the following intracellular solution (B) was used: Cs-methanesulfonate 120 mM; KCl 5 mM; CaCl_2_ 1 mM; MgCl_2_ 2 mM; EGTA 10 mM; Na_2_ATP 4 mM; Na_3_GTP 0.3 mM; lidocaine *N*-ethylbromide 5 mM; Hepes 8 mM (pH adjusted to 7.3 with KOH).

To isolate and record Ca^2+^ currents an intracellular solution composed by: CsCl, 120 mM; TEA-Cl, 20 mM; HEPES, 10 mM; EGTA, 10 mM; MgCl_2_, 2 mM; Mg-ATP, 4 mM and an extracellular solutions composed by: NaCl 120 mM; KCl 2.5 mM; NaHCO_3_ 26 mM; glucose 10 mM; MgCl_2_ 1.3 mM; CaCl_2_ 2.3 mM; NaH_2_PO_4_ 1.25 mM; TEA-Cl, 20 mM; TTX 1 (μM); 4-AP 0.1 mM were used.

When filled with the above solutions, patch pipettes had a resistance of 4–5 MΩ. Recordings were obtained with a MultiClamp 700B amplifier (Axon Instruments Molecular Devices, Sunnyvale, CA, United States), interfaced to a computer through a Digidata 1322 (Digitata, Axon Instruments Molecular Devices, Sunnyvale, CA, United States), and then acquired using Clampex 9.2 software (Molecular Devices, Palo Alto, CA, United States). Data were sampled at 20 kHz and filtered at 10 kHz.

### Drugs and Chemicals

The effects of drugs were studied upon external perfusion (bath-perfusion) of the slices. The selective activation of OtRs was performed by application of the selective OT agonist TGOT (Bachem, Bubendorf, Switzerland) in order to exclude any interference with vasopressin V1a receptors which may be present in the hippocampus (Audigier and Barberis, 1985). TGOT was generally used at the near saturating concentration of 1 μM, if not otherwise stated.

Spontaneous inhibitory postsynaptic currents (sIPSCs) were isolated by blocking the ionotropic glutamate receptors with 10 μM NBQX (2,3-dioxo-6-nitro-1,2,3,4-tetrahydrobenzo[f]quinoxaline-7-sulfonamide, Tocris Cookson, Bristol, United Kingdom) and 30 μM (RS)-CPP [(RS)-3-(2-carboxypiperazin-4-yl)-propyl-1-phosphonic acid, Tocris Cookson, Bristol, United Kingdom]. sEPSCs were isolated by blocking the ionotropic GABA_A_R with 10 μM bicuculline (Sigma-Aldrich, Oakville, ON, Canada). mIPSC were recorded during application of 1 μM TTX (tetrodotoxin, Tocris Cookson, Bristol, United Kingdom) to prevent action potential firing. OtRs were blocked using the selective antagonist SSR126768A (4-chloro-3-[(3R)-(+)-5-chloro-1-(2,4-dimethoxybenzyl)-3-methyl-2-oxo-2,3-dihydro-1H-indol-3-yl]-*N*-ethyl-*N*-(3-pyridylmethyl)-benza mide, hydrochloride, Sigma-Aldrich, Oakville, ON, Canada) at a concentration of 0.1 μM, a gift of Dr. C. Serradeil-LeGal (Sanofi-Aventis Group). Nifedipine (Sigma-Aldrich, Oakville, ON, Canada), at a concentration of 20 μM, was used to block L-type voltage-gated Ca^2+^ channels.

### Data Analysis and Statistics

Data were analyzed off-line using Clampfit 10.2 (Molecular Devices, Palo Alto, CA, United States), NeuroMatic (a collection of Igor Pro functions: WaveMetrics Inc., Oswego, OR, United States), and Microcal Origin 6.0 (OriginLab, Northampton, MA, United States).

**FIGURE 1:**
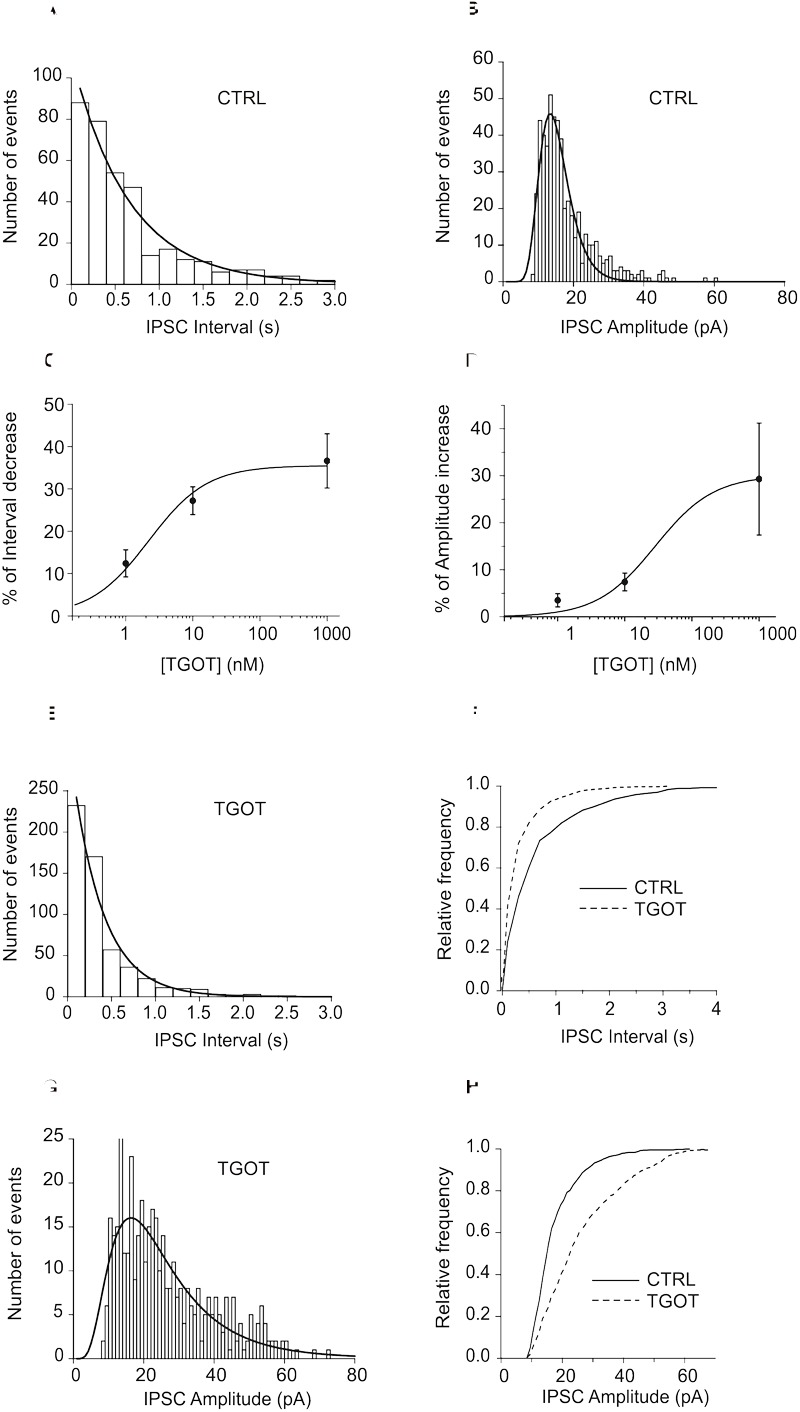
**(A)** Representative exponential distributions of the intervals of spontaneous synaptic currents under control conditions from a PYR Otr^+/+^ cell. In control saline **(A)**, the mean inter-event interval, obtained by interpolation of the interval distribution with a single exponential function, was 646 ± 49 ms (365 events). **(B)** Representative lognormal distributions of the amplitudes of spontaneous synaptic currents under control conditions from a PYR Otr^+/+^ cell. The mean log-normally distributed sIPSC amplitude was 14.7 ± 0.2 pA. **(C)** Mean dose-response relationship between the percentage of sIPSC inter-event interval decrease and TGOT concentration (*N* = 13 for 1 and 10 nM; *N* = 23 for 1 μM). **(D)** Mean dose–response relationship between the percentage of sIPSC amplitude increase and TGOT concentration (*N* = 15 for 1 nM, *N* = 18 for 10 nM and *N* = 23 for 1 μM). **(E)** In the presence of TGOT the mean inter-event interval was 351 ± 34 ms (559 events), the two interval distributions (A vs. E) being significantly different. **(F)** Cumulative distributions of the normalized frequencies vs. sIPSC intervals under control conditions (continuous line) and in the presence of 1 μM TGOT (dashed line) from the same PYR Otr^+/+^ cell as in **(A–E)**. **(G)** The mean log-normally distributed sIPSC amplitude increased to 22.4 ± 0.9 pA in the presence of TGOT. **(H)** Cumulative distributions of the normalized frequencies vs. sIPSC amplitude under control conditions (continuous line) and in the presence of 1 μM TGOT (dashed line) from the same PYR Otr^+/+^ cell as in **(B–G)**. In **(C,D)** vertical bars denote SE from the mean.

Membrane capacitance (*C*_m_) was estimated by integrating the capacitive current evoked by a -10 mV pulse, whereas input resistance (*R*_in_) was calculated from the same protocol at the end of a 20 ms pulse, when the current trace reached steady state. The postsynaptic currents were analyzed by using the automatic detection protocol within NeuroMatic and checked manually for accuracy. In all tested neurons the inter-event intervals of spontaneous and miniature postsynaptic currents (sPSCs and mPSCs, respectively) were distributed exponentially (Figure 1A) and the mean interval was computed as the time constant (τ) value of the mono-exponential function that best fitted the distribution. The amplitudes of sPSCs obeyed a lognormal distribution (Figure 1B). Accordingly, the mean amplitude was computed at the peak of the lognormal function used to fit the distribution. The mean instantaneous frequencies of synaptic currents were obtained from the reciprocal of τ. The kinetic analysis of currents was performed by measuring rise time and time constant of decay (τ_d_). Rise time represents the time that current takes to activate from 10 to 90%, whereas τ_d_ represents the time that current takes to deactivate exponentially to 37% of its peak value.

The GABA_A_R-mediated extrasynaptic currents were analyzed by applying a saturating concentration of bicuculline (10 μM), according to a well-established method (Bright and Smart, 2013). Besides blocking synaptic currents, this procedure unveiled a tonic inhibition by causing a shift in the holding current needed to clamp the membrane potential of cells at a fixed value. The quantitative analysis of the shift was performed by generating all-point histograms for 10-s-long intervals recorded under control conditions and during perfusion of bicuculline, respectively. Histograms were fitted with Gaussian functions and the mean currents, recorded before and during bicuculline perfusion, were measured at the peak of the functions. The difference between the mean currents (bicuculline – control) provided an estimation of the tonic current. The same procedure was used to reveal the modulatory effect elicited by TGOT on the tonic current, quantified by computing the difference between the mean currents (TGOT – control).

The all-point histograms were also used for the quantitative analysis of the TGOT-induced depolarization or hyperpolarization in current-clamp experiments.

The effect of TGOT on neuronal excitability was assessed during current-clamp experiments by recording voltage responses to the injection of depolarizing currents steps of increasing intensity before and after the drug administration. The quantitative analysis of the responses were performed by generating the firing rate-to-injected current (F-I) relationship, characterized by two parameters: the offset (i.e., the minimal intensity of injected current required to attain a response) and the gain (i.e., the slope of the relationship), according to literature (Brickley et al., 1996).

Statistical analysis was done with Microsoft Office Excel 2010 and Microcal Origin 6.0. All data throughout the text are expressed as mean ± SEM (standard error of the mean) and N indicates the number of neurons analyzed for each experimental procedure. Statistical significance was determined by two-tailed Student’s *t*-test or one sample *t*-test, accordingly with the type of experiment.

## Results

### Effect of TGOT on Spontaneous GABAergic Inhibitory Postsynaptic Currents Recorded From Mouse CA1 Pyramidal Neurons

GABAergic sIPSCs were whole-cell recorded from visually identified pyramidal neurons (PYR) positioned in CA1 *stratum pyramidale* of hippocampal brain slices from P17-P26 Otr^+/+^ mice and visualized by infrared microscopy.

Passive membrane properties of PYRs from Otr^+/+^ mice, such as membrane capacitance (*C*_m_) and input resistance (*R*_in_), were not significantly different from those found in literature (Martina et al., 2013) (Table 1).

**Table 1:** Passive properties of PYRs and INs in the mouse CA1 region.

	*C*_m_ (pF)	*R*_in_ (MΩ)
Otr^+/+^ PYRs	57 ± 3.2	115 ± 11
(*N* = 46)
Otr^-/-^ PYRs	51 ± 4.3	109 ± 6
(*N* = 29)
GAD67-GFP^+^ INs	41 ± 1.6	210 ± 14
(*N* = 73)

*Mean values of membrane capacitance (*C*_m_) and input resistance (*R*_in_) obtained from PYRs in Otr^+/+^ mice (*N* = 46), PYRs in Otr^-/-^ mice (*N* = 29), and from INs in GAD67-GFP^+^ (Δneo) mice (*N* = 73).*

Spontaneous inhibitory postsynaptic currents were recorded from 23 PYRs in Otr^+/+^ mice during application of gap free voltage-clamp recordings and maintaining a holding potential of 0 mV, in the presence of 10 μM NBQX and 30 μM CPP to block glutamatergic synaptic inputs. sIPSC inter-event intervals (i.e., the inverse of the instantaneous event frequency) were exponentially distributed (Figure 1A) whereas sIPSC amplitudes were distributed lognormally (Figure 1B). Recordings were performed in three conditions: (i) control saline, (ii) perfusion with the selective OT receptor (OtR) agonist TGOT at the saturating concentration of 1,000 nM (as shown in Figure 1C,D), (iii) drug wash out. To further clarify the action of TGOT on the inhibitory synaptic transmission, a quantitative analysis of the effect of the peptide was performed on the frequency and amplitude of sIPSCs. An example of the analysis of the spontaneous synaptic activity is illustrated in Figure 1, where interval distribution plots obtained under control conditions and in the presence of 1,000 nM TGOT are shown in Figure 1A,G, respectively, and the cumulative interval distributions of sIPSC in Figure 1F, whereas amplitude distribution plots in the two different conditions are shown in Figure 1B,G, respectively, and amplitude cumulative distributions in Figure 1H. Note that TGOT shifted the cumulative interval distribution toward shorter intervals (Figure 1F) and the cumulative amplitude distribution toward higher amplitudes (Figure 1H).

**FIGURE 2:**
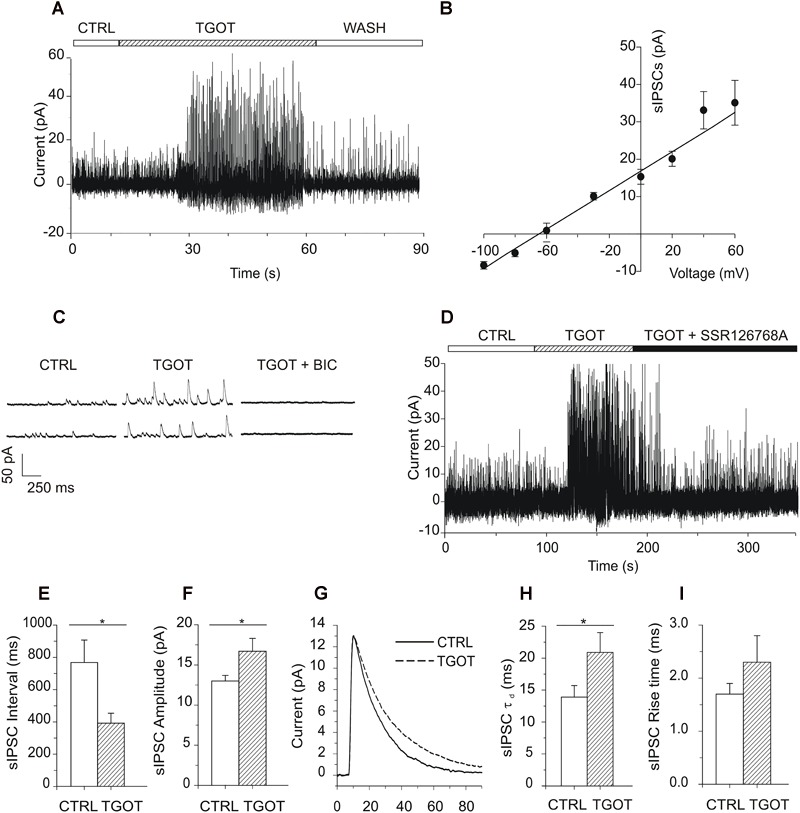
Effects of TGOT on sIPSCs in PYRs from Otr^+/+^ mice. **(A)** Sample current trace showing sIPSCs recorded during slice perfusion with control saline (CTRL), in the presence of 1 μM TGOT (TGOT) and during wash out (WASH). The upper horizontal bar indicates the time course of drug delivery. **(B)** Mean current-to-voltage relationship of the sIPSC peak amplitudes (*N* = 5), showing a reversal potential close to –60 mV. The straight line represents data point fitting. **(C)** Representative current traces of sIPSCs recorded in the presence of glutamatergic synaptic blockers at a holding potential of 0 mV under control conditions (CTRL), in the presence of 1 μM TGOT (TGOT) and during the co-administration of TGOT with the selective GABA_A_R antagonist bicuculline (10 μM) (TGOT + BIC). **(D)** Representative current trace of sIPSCs recorded in the presence of glutamatergic synaptic blockers, at a holding potential of 0 mV, under control conditions (CTRL), during TGOT administration (TGOT) and during the co-administration of TGOT with the selective murine OtR isoform antagonist SSR126768A (0.1 μM) (TGOT + SSR126768A). **(E)** Bar plots comparing the mean values of the sIPSC inter-event intervals (*N* = 23) obtained in control saline and during TGOT application (^∗^*p* < 0.05). **(F)** Bar plots comparing the mean values of the sIPSC amplitudes (*N* = 23) obtained in control saline and during TGOT application (^∗^*p* < 0.05). **(G)** Superimposed average traces of sIPSCs (lined up according to the mid-point of their rise times), recorded in the presence of glutamatergic synaptic blockers, at a holding potential of 0 mV, under control conditions (continuous line, averaging of 25 traces) and during TGOT application (dashed line, averaging of 168 traces whose amplitude was normalized to that of control). **(H)** Bar plots comparing the mean values (*N* = 4) of the sIPSC time constant of decay (τ_d_) obtained under control conditions and during TGOT application (^∗^*p* < 0.05). **(I)** Bar plots comparing the mean values (*N* = 4) of the sIPSC rise time obtained under control conditions and during TGOT application. Vertical bars in **(B,E,F,H,I)** indicate SE from the mean.

The Nernst’s derived equilibrium potential for Cl^-^ was -63 mV and the sIPSCs measured at 0 mV appear as outward-going currents (Figure 2A). A reversal potential of -64 mV was calculated from the I–V relationship (Figure 2B) and confirmed that the measured currents were carried by chloride ions. Further, as shown in the sample traces of Figure 2C, the events could be abolished by bicuculline at 10 μM (*N* = 3), confirming that the synaptic currents were mediated via GABA_A_Rs.

In 23 out of 23 PYRs TGOT increased the frequency and the amplitude of sIPSCs in a reversible way, as shown by the example trace in Figure 2A, in agreement with previous observations in rat (Zaninetti and Raggenbass, 2000). The delay between the beginning of TGOT application and the onset of the effect of TGOT was probably due to the relatively slow diffusion of the peptide in the bath extracellular saline and within the hippocampal slice. In addition, the effect of TGOT on sIPSCs was suppressed by the selective OtR antagonist SSR126768A, added to the perfusion solution at 0.1 μM (Figure 2D, *N* = 3), confirming that OtRs were selectively involved.

On average we measured an inter-event interval of 760 ± 137 ms and of 388 ± 61 ms under control conditions and during application of 1,000 nM TGOT, respectively (Figure 2E, *p* < 0.02). A significant increase in the sIPSC amplitude was measured as well: on average it was 13.0 ± 0.7 pA and 16.7 ± 1.6 pA under control conditions and during application of 1,000 nM TGOT, respectively (Figure 2F, *p* < 0.04). Both sIPSC inter-event interval decrease and amplitude increase mediated by TGOT application turned out to be concentration dependent with IC_50_ of 2.2 nM and 28.4 nM, respectively (Figure 1C,D).

Interestingly, an effect of TGOT was evident also on the deactivation kinetics but not on the rise time of sIPSCs (Figure 2G). Indeed, TGOT caused a significant increase in the time constant of decay (τ_d_) of sIPSCs in four out of four examined PYRs (*p* < 0.05): on average τ_d_ was 13.9 ± 1.8 ms under control conditions and 20.9 ± 3.1 ms during application of TGOT (Figure 2H). On the other hand, rise time was not significantly altered by TGOT: on average rise time was 1.7 ± 0.2 ms under control conditions and 2.3 ± 0.5 ms during application of TGOT (Figure 2I). It is remarkable that an increase in the time course of sIPSC deactivation could significantly prolong the hyperpolarized state of the cell, in particular if the time constant of sIPSC decay would be equal to or greater than the plasma membrane time constant τ_m_. Indeed, τ_m_ was on average 6.7 ms in PYRs.

**FIGURE 3:**
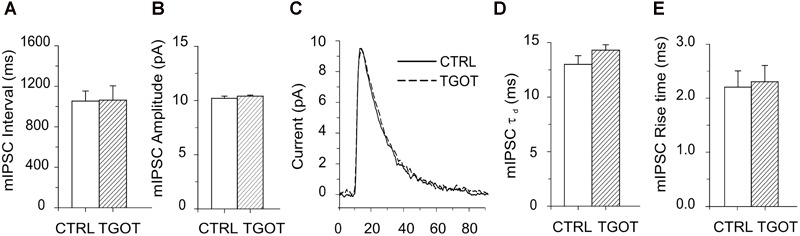
Effect of TGOT on mIPSCs in PYRs from Otr^+/+^. **(A)** Bar plots comparing the mean mIPSC inter-event intervals obtained from three CA1 PYRs under control conditions and during perfusion with TGOT. **(B)** Bar plots comparing the mean mIPSC amplitudes obtained from three CA1 PYRs under control conditions and during perfusion with TGOT. **(C)** Superimposed average traces of mIPSCs (lined up according to the mid-point of their rise times) recorded from a CA1 PYR. Currents were recorded under control conditions (CTRL: continuous line, average of races) and during the application of TGOT (TGOT: dashed line, average of 37 traces), and were normalized to the control. **(D)** Bar plots comparing the mean mIPSC time constant of decay (τ_d_) obtained from three CA1 PYRs under control conditions and during TGOT administration. **(E)** Bar plots comparing the mean mIPSC rise time obtained from three CA1 PYRs under control conditions and during TGOT administration. mIPSCs were always recorded at a holding potential of 0 mV, in the presence of glutamatergic synaptic blockers and TTX (1 μM), in order to prevent action potential firing. In all bar plots the vertical bars denote SE from the mean.

The TGOT-induced increase in the sIPSC amplitude and frequency is likely due to the peptide acting on OtRs expressed by GABAergic INs. This action of TGOT could be exerted either at the somatodendritic membrane of the inhibitory cells, in an action potential-dependent way, and/or at their axon terminals, by affecting the probability of the release of synaptic vesicles in an action potential-independent way. To clarify this point, the effect of TGOT was examined in the presence of 1 μM TTX in the extracellular saline on miniature, action potential-independent, IPSCs (mIPSCs). TGOT did not cause any significant modification of either the mIPSC amplitude or inter-event interval in any of the three PYRs tested. The mean interval obtained from the best exponential fit of the data (*N* = 3) was 1,054 ± 99 ms in control conditions and 1,063 ± 141 ms in the presence of TGOT (Figure 3A). The mIPSC amplitudes had a mean value of 10.2 ± 0.2 pA in control conditions and 10.4 ± 0.1 pA (*N* = 3) in the presence of TGOT (Figure 3B).

All these observations, and in particular the increase in the amplitude and frequency of sIPSCs but not of mIPSCs during TGOT application, suggest that the peptide enhanced the inhibitory transmission in the CA1 region of the hippocampus by acting on OtRs located on the somatodendritic membrane of GABAergic INs rather than on their axon terminals.

Should also be noted that neither the rise time nor the decay time constant of mIPSC were significantly affected by TGOT (Figure 3C–E): on average rise time was 2.2 ± 0.3 ms in control conditions and 2.3 ± 0.3 ms during perfusion of TGOT (*N* = 3), while τ_d_ was 13.0 ± 0.8 ms and 14.3 ± 0.5 ms, respectively (*N* = 3).

To further investigate the effects of TGOT on the GABAergic activity onto CA1 PYRs and the importance of expression of selective OtRs, we analyzed neurons from Otr^-/-^ mice (Sala et al., 2011).

Passive membrane properties of PYRs in Otr^-/-^ mice, such as *C*_m_ and *R*_in_, were not significantly different from those measured in Otr^+/+^ mice (Table 1). Thus, although Otr^-/-^ mice exhibit numerous behavioral deficits (Lee et al., 2008; Sala et al., 2011), the intrinsic passive electrical properties of their hippocampal PYRs are not significantly altered.

**FIGURE 4:**
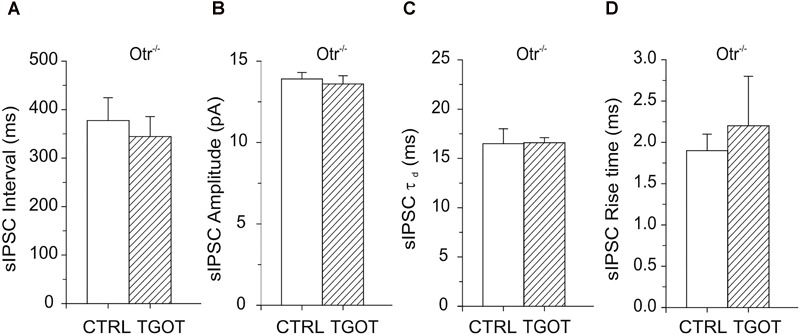
Effects of TGOT on PYRs in Otr^-/-^ mice. **(A)** Bar plots comparing the mean values of the sIPSC inter-event intervals (*N* = 15) in Otr^-/-^ mice obtained under control conditions and during TGOT application. **(B)** Bar plots comparing the mean values of the sIPSC amplitudes (*N* = 15) in Otr^-/-^ mice obtained in control saline and during TGOT application. **(C)** Bar plots comparing the mean values (*N* = 4) of the sIPSC time constant of decay (τ_d_) in Otr^-/-^ mice obtained under control conditions and during TGOT application. **(D)** Bar plots comparing the mean values of the sIPSC rise time (*N* = 4) in Otr^-/-^ mice obtained under control conditions and during TGOT application. Vertical bars indicate SE from the mean.

As expected, no significant effect of TGOT was measured either on sIPSC interval (378 ± 47 ms in control against 345 ± 41 ms with TGOT, *N* = 15), amplitude (13.9 ± 0.4 pA in control against 13.6 ± 0.5 pA with TGOT, *N* = 15), rise time (1.9 ± 0.2 ms in control against 2.2 ± 0.6 ms with TGOT, *N* = 4) or time constant of decay (16.5 ± 1.5 ms in control against 16.6 ± 0.5 ms with TGOT, *N* = 4) in Otr^-/-^ PYRs, as shown in Figure 4.

### Effect of TGOT on Spontaneous Excitatory Postsynaptic Currents in PYRs From Otr^+/+^ and Otr^-/-^ Mice

**FIGURE 5:**
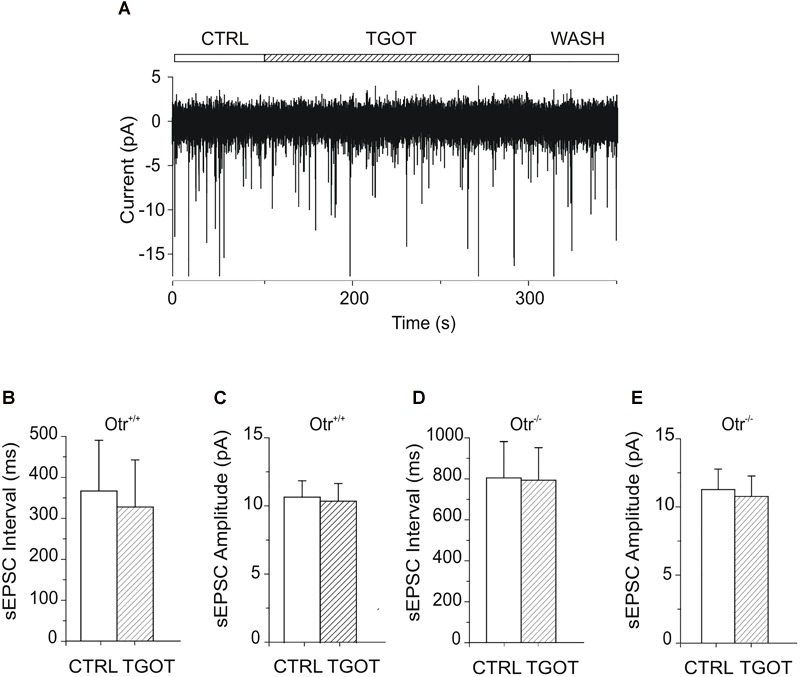
Effect of TGOT on sEPSCs in PYRs from Otr^+/+^ and Otr^-/-^ mice. **(A)** Representative current trace of sEPSCs recorded from a CA1 PYR of an Otr^+/+^ mouse in the presence of the GABAergic synaptic blocker bicuculline (10 μM), at a holding potential of –70 mV, under control conditions (CTRL), during TGOT administration (TGOT) and during drug wash out (WASH). **(B)** Bar plots comparing the mean sEPSC interval (*N* = 7) obtained in control saline and during TGOT administration from PYRs in Otr^+/+^ mice. **(C)** Bar plots comparing the mean sEPSC amplitude (*N* = 7) obtained in control saline and during TGOT administration from PYRs in Otr^+/+^ mice. **(D)** Bar plots comparing the mean sEPSC interval (*N* = 8) obtained in control saline and during TGOT administration from PYRs in Otr^-/-^ mice. **(E)** Bar plots comparing the mean sEPSC amplitude (*N* = 8) obtained in control saline and during TGOT administration from PYRs in Otr^-/-^ mice. In all bar plots the vertical bars denote SE from the mean.

A possible effect of TGOT on spontaneous excitatory postsynaptic currents (sEPSCs) in Otr^+/+^ and Otr^-/-^ PYRs of the mouse CA1 region was examined as well. sEPSCs were recorded from seven PYRs in Otr^+/+^ mice during gap-free voltage-clamp recordings at a holding potential of -70 mV. Experiments were performed in control conditions, during perfusion of 1 μM TGOT, and following drug wash out. To exclude the activation of GABAergic receptors, GABA_A_R blocker bicuculline was present in the perfusion solution at 10 μM. Intra- and extracellular saline had the physiological concentrations of ions, so that the reversal potential of AMPA currents was about 0 mV and, thus, the sEPSCs measured at -70 mV should appear as inward-going currents (Figure 5A).

In contrast to what observed for GABAergic currents, no significant effect of TGOT could be detected on either the sEPSC inter-event interval or amplitude in any of the seven Otr^+/+^ and of the eight Otr^-/-^ PYRs tested (Figure 5A–E). In Otr^+/+^ neurons the mean sEPSC interval was 363 ± 123 ms in control conditions and 324 ± 115 ms in the presence of TGOT, while the sEPSC mean amplitude was 10.7 ± 1.2 pA in control conditions and 10.4 ± 1.3 pA in the presence of the peptide. In Otr^-/-^ neurons the mean inter-event intervals were 804 ± 178 ms in control saline and 793 ± 159 ms in the presence of TGOT, whereas the mean amplitude values were 11.2 ± 1.5 pA and 10.7 ± 1.5 pA in controls and during peptide application, respectively.

### Effect of TGOT on the Extrasynaptic Transmission in CA1 Field

**FIGURE 6:**
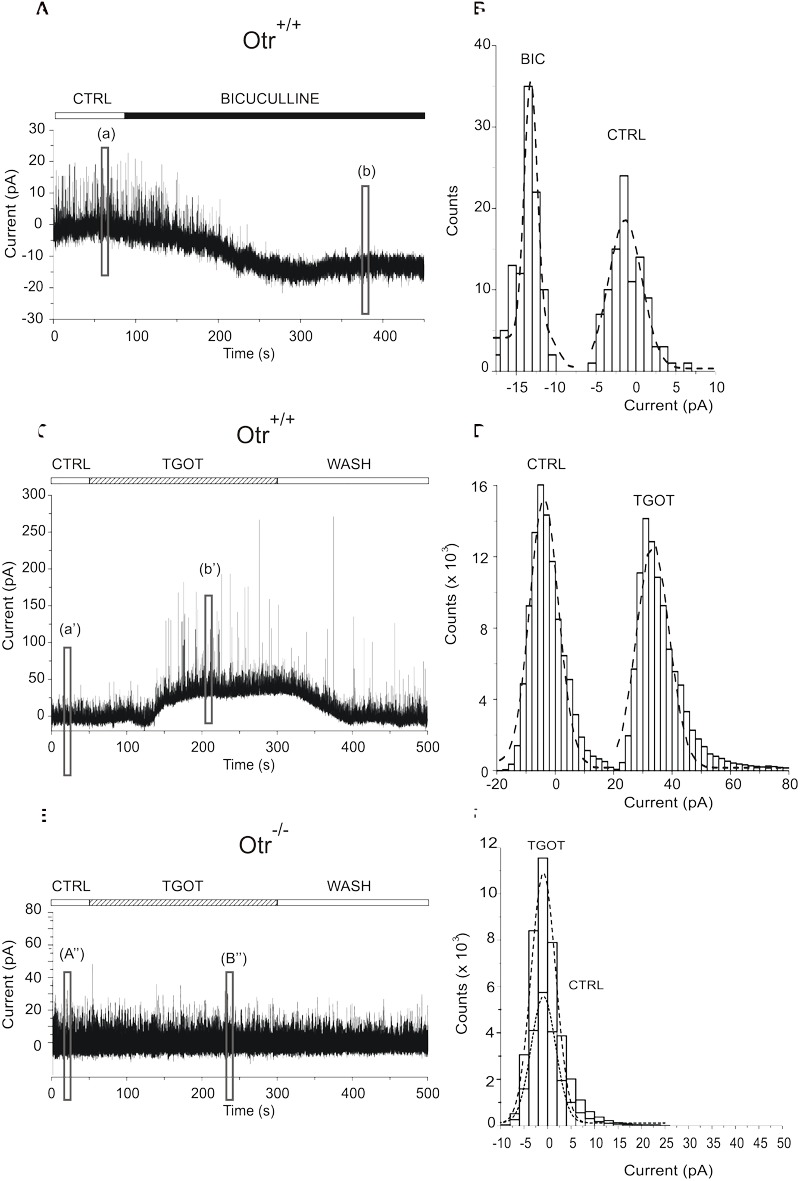
Effect of TGOT on the extrasynaptic transmission in CA1 PYR. **(A)** Representative current trace of sIPSCs recorded from a PYR of an Otr^+/+^ mouse in the presence of glutamatergic synaptic blockers, at a holding potential of 0 mV, in control saline (CTRL) and during perfusion with bicuculline. (a) and (b) boxes indicate the 10-s-long intervals used for the construction of the all-point histograms in **(B)**. The upper horizontal bar indicates the time course of drug delivery. **(B)** Representative all-point histograms for the intervals shown in **(A)**; dashed curves were obtained by fitting data points with Gaussian functions. The peaks of dashed curves represent the mean current values recorded before (CTRL) and during bicuculline (BIC) perfusion. The difference between the mean currents (BIC – CTRL) provides the inward shift, revealing the presence of tonically active currents mediated by extrasynaptic GABA_A_Rs. **(C)** Representative current trace of sIPSCs recorded from a PYR of an Otr^+/+^ mouse in the presence of glutamatergic synaptic blockers, at a holding potential of 0 mV, in control saline (CTRL), during perfusion with TGOT (TGOT) and during wash out (WASH). (a’) and (b’) boxes indicate the 10-s-long intervals used for the construction of the all-point histograms in **(D)**. **(E)** Representative current trace of sIPSCs recorded from a PYR of an Otr^-/-^ mouse in the presence of glutamatergic synaptic blockers, at a holding potential of 0 mV, in control saline (CTRL), during perfusion with TGOT (TGOT) and during wash out (WASH). (a”) and (b”) boxes indicate the 10-s-long intervals used for the construction of the all-point histograms in **(F)**.

The data described so far indicate that TGOT modulates the inhibitory transmission in CA1 *stratum pyramidale*. In the hippocampus, the main source of inhibition onto PYRs is represented by GABAergic INs (Freund and Buzsáki, 1996). GABA exerts its action by binding mainly to GABA_A_Rs expressed on the synaptic membrane of PYRs (‘phasic’ inhibition) (Farrant and Nusser, 2005) or can escape from the synaptic cleft by spillover and activate GABA_A_Rs located in perisynaptic (Wei et al., 2003) or extrasynaptic (Nusser et al., 1998) sites (‘tonic’ inhibition). We therefore tested the possibility that, besides an effect on phasic inhibitory currents, TGOT could also modulate the tonic inhibition mediated by activation of extrasynaptic GABA_A_Rs on CA1 PYRs. To this purpose, the measurement of this GABAergic tonic current has been performed according to a standard and widely used method consisting in the measurement of the shift of the holding current induced by cell perfusion with a specific drug (Kaneda et al., 1995; Wall and Usowicz, 1997; Bright and Smart, 2013). First, we assessed the expression of tonic GABAergic currents in CA1 PYRs. Activation of extrasynaptic GABAergic currents was tested in five Otr^+/+^ PYRs during application of gap free protocols in voltage-clamp at a holding potential of 0 mV. Experiments were performed under control conditions and during perfusion with 10 μM bicuculline to block both synaptic and extrasynaptic GABA_A_Rs (Wlodarczyk et al., 2013). As expected, bicuculline blocked all sIPSCs and caused an inward shift of the holding current required to clamp PYR membrane potential at 0 mV (Figure 6A), consistently with the abolition of GABA_A_R-mediated tonic currents, as described in literature (Bright and Smart, 2013). It must be noted that the current shift was inward since E_Cl_ (-63 mV) was close to the resting membrane potential and the holding potential was 0 mV. A quantitative analysis of current traces, performed by creating all-point histograms (Figure 6B), revealed a mean current shift of -12.3 ± 2.7 pA (*N* = 5). Subsequently, the putative action of TGOT on tonic GABAergic currents was assessed in 25 PYRs. An example of how TGOT influenced the holding current is shown in Figure 6C: the peptide caused a reversible current shift, whose direction was opposite to that described for the GABA_A_R antagonist bicuculline. This outward shift is consistent with a positive modulation of tonic currents mediated by extrasynaptic GABA_A_Rs. The quantitative analysis of the traces, performed by generating all-point histograms (Figure 6D), revealed an outward current shift in 14 out of 25 PYRs examined: on average the shift was +46.4 ± 14.9 pA. This effect of TGOT was undoubtedly related to the presence of OtRs, since the agonist was not able to cause any significant change in the holding current in Otr^-/-^ mice (*N* = 15/15) (Figure 6E,F).

Overall, our results indicate that TGOT is able to increase not only the phasic (synaptic) but also the tonic (extrasynaptic) GABA_A_R-mediated inhibition onto PYRs.

### Effect of TGOT on the Membrane Potential of CA1 GABAergic Interneurons

**FIGURE 7:**
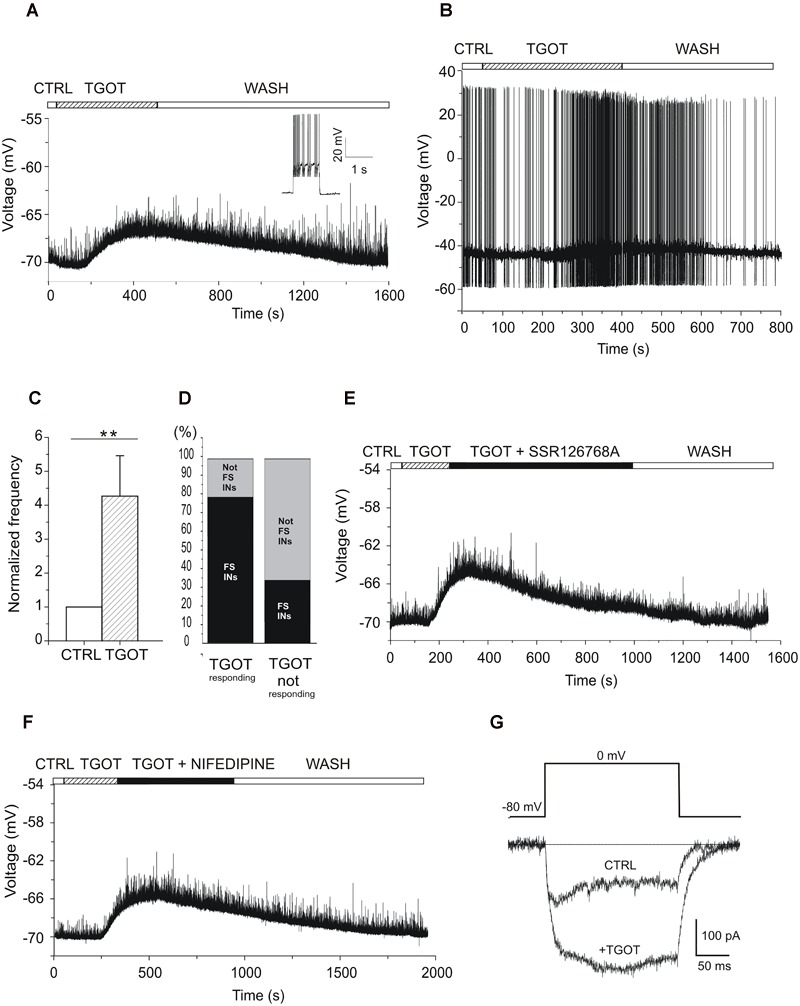
Effect of TGOT on the membrane potential and firing frequency of CA1 INs. **(A)** Representative voltage trace recorded from a GABAergic IN (inset: the stuttering fast-spiking firing modality) without synaptic blockers, at a membrane potential of –70 mV, under control conditions (CTRL), during TGOT application (TGOT) and during wash out (WASH). The upper horizontal bar indicates the time course of drug delivery. TGOT caused a depolarizing response. **(B)** Representative voltage trace recorded from a GABAergic IN without synaptic blockers, at spike threshold, under control conditions (CTRL), during TGOT application (TGOT) and during wash out (WASH). TGOT caused depolarization together with an increase in firing rate. **(C)** Bar plots comparing the firing frequency at threshold, normalized to control, during TGOT application. TGOT caused a significant increase in spike frequency (*N* = 18, ^∗∗^*p* < 0.005). The vertical bars denote SE from the mean. **(D)** Bar plot indicating the TGOT-responding (left column) and TGOT-not responding (right column) cells. The majority (77%) of TGOT responding cells were fast spiking INs (left column-black), whereas the majority (67%) of TGOT not responding cells were not fast spiking INs (right column-light gray). **(E)** Representative voltage trace recorded from a stuttering fast-spiking GABAergic IN without synaptic blockers, at the membrane potential of –70 mV, under control conditions (CTRL), during TGOT administration (TGOT), during co-application of TGOT with the selective antagonist for the OtR murine isoform SSR126768A (0.1 μM) (TGOT + SSR126768A) and during wash out (WASH). **(F)** Representative voltage trace recorded from a GABAergic IN without synaptic blockers, at the membrane potential of -70 mV, in control conditions (CTRL), during TGOT administration (TGOT), and during co-administration of TGOT and the selective L-type Ca^2+^ channel blocker nifedipine (20 μM) (TGOT + NIFEDIPINE). **(G)** Representative isolated Ca^2+^-current traces recorded at 0 mV in control condition (CTRL) and following TGOT administration (+TGOT) in the presence of glutamatergic and GABAergic synaptic blockers. **(E)** Bar plots comparing the mean firing frequency at threshold, normalized to control, obtained from six CA1 PYRs under control conditions and during perfusion with TGOT. Vertical bars in **(C,E)** denote SE from the mean.

To elucidate the neuronal circuit involved in this oxytocinergic action in the CA1 field of mouse hippocampus, we evaluated the effect of TGOT directly on the membrane potential of CA1 *stratum pyramidale* GABAergic INs, identified in GAD67-GFP^+^ (Δneo) mice because of their GFP-dependent fluorescence. The nature of these cells was confirmed by the higher input resistance (*R*_in_, *p* < 0.001) and lower membrane capacitance (*C*_m_, *p* < 0.001) compared to PYRs (Table 1). The voltage responses were evaluated in current-clamp conditions from 73 INs following application of gap-free protocols before and during TGOT application. Experiments were performed both without glutamatergic and GABAergic synaptic blockers (*N* = 58) and during synaptic isolation (*N* = 15). Our results show that in absence of synaptic blockers about 50% of the overall tested INs at -70 mV (*N* = 31/58) responded to TGOT with a sustained depolarization (Figure 7A), whereas the remaining fraction of cells did not respond at all to the agonist. The mean depolarization, calculated using all-point histograms, was +6.2 ± 0.8 mV. A subset of 18 cells responding to TGOT at -70 mV was tested also at threshold: in all of them TGOT caused a depolarizing response (on average: +2.5 ± 0.3 mV) (Figure 7B) together with a significant increase in the firing rate by a factor of 2.7 ± 0.9 (Figure 7C, *p* < 0.005). Based on their firing mode GABAergic INs have been subdivided in Fast- (FSn = 33/58) and not fast spiking (not FS; *n* = 25/58) neurons. Interestingly, the majority of INs responding to TGOT were stuttering fast-spiking neurons (77% *n* = 24/31), characterized by a high frequency firing rate (inset to Figure 7A), whereas the majority of not-responding INs were not fast spiking cells (67% *n* = 18/27) (Figure 7D), in agreement with observations reported in literature on rats (Owen et al., 2013). Similar results were obtained in the presence of glutamatergic and GABAergic synaptic blockers: indeed, 8 stuttering fast-spiking INs out of 15 GABAergic cells tested at -70 mV displayed a sustained depolarization (on average: +6.3 ± 1.1 mV) during co-perfusion of TGOT, NBQX, CPP, and bicuculline. Among them a subset of five stuttering fast-spiking cells responding to TGOT was tested also at threshold under the same conditions: all of them responded to the agonist with a depolarization (on average: +3.4 ± 1.0 mV) together with a significant increase (*p* < 0.05) in the firing rate by a factor of 3.4 ± 1.1.

This observation that the TGOT-induced depolarization was elicited despite synaptic blockade suggests a direct binding of TGOT to OtRs expressed in those cells and not a secondary effect of TGOT on upstream neurons. To corroborate this conclusion, we recorded voltage responses from stuttering fast-spiking INs during co-administration of TGOT and the selective OtR antagonist SSR126768A (0.1 μM) (Figure 7E). In the totality of the examined cells (*N* = 6/6) the TGOT-mediated depolarization was completely abolished in the presence of the antagonist, supporting the idea that TGOT directly activates OtRs expressed in stuttering fast-spiking GABAergic INs.

After the identification of the subpopulation of GABAergic INs expressing OtRs and directly responding to TGOT, we preliminarily explored the putative ionic mechanism involved in the TGOT-induced depolarization of those cells. OtRs are defined ‘promiscuous’ G protein-coupled receptors (GPCRs) because of their ability to bind different G protein subtypes, such as *G*_i_ or *G*_q/11_ (Chini et al., 2008; Manning et al., 2008). In general, activation of *G*_q/11_ can lead to the phosphorylation of different intracellular substrates, including L-type voltage-gated Ca^2+^ channels (Weiss et al., 2012; Satin, 2013). To test a possible involvement of voltage-gated Ca^2+^ channels in the TGOT-mediated pathway, we first recorded voltage responses of GABAergic INs in the presence of TGOT and then during the co-application of TGOT and nifedipine (20 μM), a selective blocker of L-type Ca^2+^ channels. Nifedipine was able to abolish the TGOT-induced effect in 13 out of 15 examined INs (Figure 7F), suggesting that L-type Ca^2+^ channel up-modulation might play a role in the membrane depolarization induced by OtR activation by TGOT. To directly confirm an oxytocinergic up-modulation of those channels, Ca^2+^-channel currents were isolated and recorded at 0 mV before and during perfusion with TGOT (*n* = 4). Our data showed a TGOT-induced increase of 34.6% ± 16.2 of the high voltage activated Ca^2+^ currents elicited in GABAergic INs (Figure 7G).

### Effect of TGOT on the Membrane Potential of CA1 Pyramidal Neurons

**FIGURE 8:**
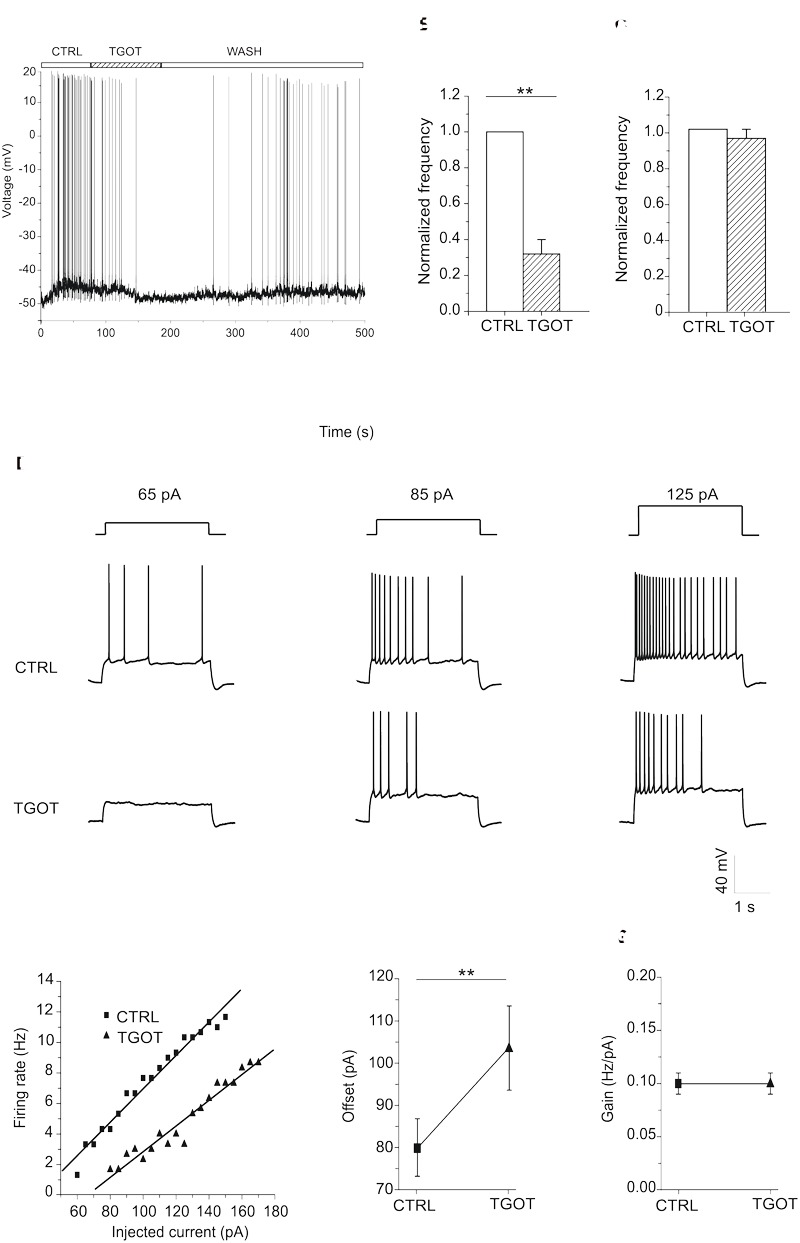
Effect of TGOT on the membrane potential and firing frequency of CA1 PYRs. **(A)** Representative voltage trace recorded from a PYR, without synaptic blockers and starting from spike threshold, in control saline (CTRL), during TGOT administration (TGOT) and during drug wash out (WASH). TGOT caused hyperpolarization together with a decrease in firing rate. **(B)** Bar plots comparing the firing frequency at threshold, normalized to control, obtained from 10 experiments under control conditions and during TGOT administration. The vertical bars denote SE from the mean. **(C)** Bar plots comparing the mean firing frequency at threshold, normalized to control, obtained from six CA1 PYRs in the presence of glutamatergic and GABAergic synaptic blockers under control conditions and during perfusion with TGOT. Vertical bars denote SE from the mean. **(D)** Representative voltage traces recorded from PYRs, starting from –70 mV, in response to the injection of depolarizing current steps of increasing amplitude (65, 85, and 125 pA, respectively, from left to right), in control conditions (CTRL, upper traces) and in the presence of TGOT (TGOT, lower traces). The firing frequency obtained during TGOT administration was always lower than that obtained under control conditions. **(E)** Firing rate-to-injected current (F-I) relationship referred to the traces of **(D)**. Data points (squares: CTRL; triangles: TGOT) were fitted with linear regression functions. **(F)** Comparison of the offset mean values obtained in seven experiments under control conditions and during perfusion of TGOT. The agonist caused a significant increase in the offset (^∗∗^*p* < 0.005). **(G)** Comparison of the gain mean values between control conditions and during perfusion of TGOT (*N* = 7).

Next, we examined the effects of TGOT on the membrane potential of CA1 PYRs (Figure 8). The action of the agonist was tested in 34 Otr^+/+^ PYRs in current-clamp mode, both without glutamatergic and GABAergic synaptic blockers (*N* = 20) and during synaptic isolation (*N* = 14). Protocols were applied either with a membrane potential of -70 mV or starting from spike threshold. Our results show that both in the absence (*N* = 7) and in the presence (*N* = 8) of synaptic blockers the totality of PYRs did not respond at all to TGOT when starting from a membrane potential of -70 mV (data not shown). Interestingly, our results show that at spike threshold in 10 out of 15 examined PYRs TGOT caused a hyperpolarizing response (on average: -3.0 ± 0.4 mV) together with a significant decrease by a factor of 0.7 ± 0.1 (*p* < 0.005) in the firing rate in the absence of synaptic blockers (Figure 8A,B). By contrast, no TGOT-mediated hyperpolarization and changes in the firing rate could be induced in six out of six PYRs during application of synaptic blockers (Figure 8C). This important result suggests that the hyperpolarizing effect of TGOT on PYRs is not direct but closely dependent on the activation of OtRs in upstream GABAergic neurons.

Since the main consequence of hyperpolarization is a reduction in cell excitability (Farrant and Nusser, 2005), we subsequently tested the effect of TGOT directly on the capability of PYRs to generate action potentials.

The effect of the OtR agonist on cell excitability was evaluated in seven Otr^+/+^ PYRs, by recording the voltage response to depolarizing injected current of increasing amplitude. Current stimuli were applied starting from a membrane potential of -70 mV both under control conditions and during perfusion with TGOT. As shown in the example traces of Figure 8D, TGOT caused a decrease of the firing frequency when compared to controls. The quantitative analysis of the responses was carried out by plotting the firing rate measured at each value of injected current versus the injected current itself (F-I plots). In the totality of tested PYRs the F-I relationship obtained during perfusion with TGOT was shifted to the right when compared to the control one (Figure 8E), indicating that TGOT was able to cause a significant increase in the offset (i.e., the minimal intensity of injected current required to get a response). Indeed, the average offset was 80 ± 7 pA in control conditions and 104 ± 10 pA in the presence of TGOT (Figure 8F, *p* < 0.05). By contrast, the gain (i.e., the slope of the F-I relationship) was not significantly modulated by TGOT (Figure 8G, 0.10 ± 0.01 Hz/pA in control conditions and 0.10 ± 0.01 Hz/pA during the application of TGOT). As described in literature, an increase in the offset is attributable to an increase in tonically active inhibitory currents, causing a persistent reduction in the input resistance and thereby in cell excitability (Mitchell and Silver, 2003).

## Discussion

A set of experiments described in the present work demonstrates that the selective OtR agonist TGOT can strongly enhance the amplitude and frequency of GABAergic sIPSCs in PYRs from Otr^+/+^ mice, but it has no effect on fast glutamatergic synaptic transmission. These results confirm and extend in mouse previous data obtained in rat and showing an OT-mediated increase of the GABAergic inhibition on hippocampal CA1 pyramidal neurons (Mühlethaler et al., 1983, 1984; Raggenbass et al., 1989; Zaninetti and Raggenbass, 2000; Owen et al., 2013).

By taking advantage of the availability of a knockout mouse for OtR (OtR^-/-^) and of a heterozygous GAD67-GFP^+^ knock-in mouse, the second part of the paper was committed to demonstrate for the first time that the TGOT-mediated increase of GABAergic inhibition in pyramidal cells is due to a direct OtR-mediated excitation of a specific subpopulation of GABAergic INs. The action of the peptide is exerted throughout an up modulation of nifedipine-sensitive, voltage-gated calcium channels. Furthermore, we have shown that in PYRs TGOT affects the deactivation kinetics of GABAergic sIPSCs and modulates both phasic and tonic GABA_A_R-mediated Cl^-^ currents.

### Effect of TGOT on Spontaneous GABAergic IPSCs Recorded From Mouse CA1 Pyramidal Neurons

The reversible increase in sIPSC frequency and amplitude produced by TGOT application on mouse hippocampal CA1 PYRs is in agreement with previous data obtained in rat (Zaninetti and Raggenbass, 2000). The effects elicited by TGOT on the GABA_A_Rs mediated inhibitory transmission are closely related to the activation of OtRs, widely described in CA1 area (Tribollet et al., 1988; Yoshimura et al., 1993; Barberis and Tribollet, 1996; Campbell et al., 2009; Ophir et al., 2012). Furthermore, the absence of any effect of TGOT in Otr ^+/+^ mice during application of an antagonist selective for the murine isoform of OtRs and in Otr^-/-^ mice definitely demonstrates the involvement of OT selective receptors for this outcome.

Interestingly, we have shown that besides its effects on interevent interval and amplitude of sIPSCs, TGOT also causes a significant increase in the sIPSC deactivation kinetics. It is noteworthy that an increase in the time course of sIPSP decay could prolong the hyperpolarized state of the cell; this is particularly true if the sIPSC time constant of decay τ_d_ is equal to or greater than the plasma membrane time constant τ_m_, as actually occurs in PYRs. In general, the activation and deactivation kinetics of sIPSCs depend on the rate of GABA clearance in the synaptic cleft and on the biophysical properties of GABA_A_Rs (Overstreet et al., 2000). In general, the decay of sIPSC is dominated by GABA_A_R deactivation, whose speed is greatly affected by their subunit composition and location (Jones and Westbrook, 1995). For example, in the hippocampus, α5-containing GABA_A_Rs located in a perisynaptic position deactivate ∼3-fold slower than synaptic receptors (Prenosil et al., 2006). Accordingly, the slower deactivation kinetics we measured in a fraction of sIPSCs during TGOT application could be attributable to the activation of perisynaptic receptors. Generally, perisynaptic GABA_A_Rs are activated by spillover that is proportional to the intensity of the firing activity of the presynaptic terminal (Farrant and Nusser, 2005). We have shown that TGOT leads to the activation of perisynaptic GABA_A_Rs by modulating the firing activity of GABAergic INs synaptically connected to PYRs. Consistent with this hypothesis no significant changes were detected in inter-event interval, amplitude, and time constant of decay of mIPSCs recorded from PYRs during TGOT application.

### Effect of TGOT on the Extrasynaptic Transmission in CA1 Field

The data described so far indicate that TGOT is able to modulate the inhibitory transmission mediated by GABA_A_Rs located at synaptic or perisynaptic sites on PYRs. In hippocampal PYRs, in addition to synaptic and perisynaptic GABA_A_Rs, the presence of GABA_A_Rs located in extrasynaptic sites has been reported as well (Banks and Pearce, 2000; Scimemi et al., 2005; Mortensen and Smart, 2006). As for perisynaptic receptors, peculiar biophysical properties are associated also to the subunits of these receptors: in the hippocampus, α5-containing extrasynaptic GABA_A_Rs have a higher affinity for GABA (Böhme et al., 2004) and desensitize slower (Caraiscos et al., 2004) than synaptic receptors, making them more responsive to GABA molecules diffusing in the extracellular space (Bright and Smart, 2013) and causing tonic neuronal inhibition (Banks and Pearce, 2000; Scimemi et al., 2005; Mortensen and Smart, 2006; Pavlov et al., 2009). By measuring an inward shift in the holding current recorded from PYRs in the presence of GABA_A_R antagonist bicuculline we demonstrated the presence of GABAergic tonic current that is strictly and directly related to the firing activity of pre-synaptic GABAergic neurons and is produced by the activation of GABA_A_Rs located in the extrasynaptic sites (Farrant and Nusser, 2005). Indeed, during TGOT application we observed an outward shift in the holding current recorded from PYRs supporting the hypothesis that TGOT could increase the magnitude of extrasynaptic GABA_A_R-mediated currents onto PYRs by inducing GABAergic INs to release a larger amount of neurotransmitter in the synaptic cleft. Overall, our results strongly suggest that TGOT is able to increase not only the phasic (synaptic) but also the tonic (extrasynaptic) GABA_A_R-mediated inhibition onto CA1 PYRs.

### Effect of TGOT on the Membrane Potential of CA1 GABAergic Interneurons

To investigate in detail the source of that increased inhibition onto PYRs we tested the effect of TGOT directly on the membrane potential of CA1 GABAergic INs and we found that about 50% of INs examined at -70 mV responded to TGOT with a sustained depolarization. Interestingly and in agreement with what reported by Owen et al. (2013) the majority of responding INs were stuttering fast-spiking cells, characterized by high frequency firing, whereas the majority of not-responding INs were not fast-spiking cells. The TGOT responding INs when examined in synaptic isolation and at their spike threshold displayed both a depolarizing response to TGOT and a significant increase in the firing rate. These data strongly suggest that the facilitation in the GABA_A_R-mediated transmission elicited by TGOT is due to an increase in firing activity mediated by activation of OtRs expressed mainly in a specific subpopulation of GABAergic INs.

This result is corroborated by the observation that the selective blockade of OtRs completely abolished the TGOT-induced depolarizing response.

Next, we tried to identify the putative ionic mechanism involved in the TGOT-induced depolarization in the sub-population of GABAergic INs. OtRs are ‘promiscuous’ GPCRs, being able to activate different G protein subtypes (Chini et al., 2008). OtRs display high affinity for *G*_q/11_ (Qian et al., 1998; Gimpl and Fahrenholz, 2001), whose activation stimulates phospholipase Cβ to produce DAG that in turn activates a protein kinase C causing phosphorylation of different target proteins (Gimpl and Fahrenholz, 2001) including L-type voltage-gated Ca^2+^ channels (Yang and Tsien, 1993; Weiss et al., 2012; Satin, 2013). This could suggest L-type Ca^2+^current up-modulation as a putative mechanisms to increase cell depolarization in those cells. Indeed, we have shown that TGOT application can up-modulate voltage-gated Ca^2+^ currents. Furthermore, nifedipine, a selective L-type Ca^2+^ channel blocker, at a concentration of 20 mM completely abolished the depolarization and the increase in the firing rate elicited by TGOT on GABAergic INs. It should be pointed out that we performed our recordings starting from spike threshold (∼-50 mV), a value at which the classical L-type Ca^2+^ channels are not active yet. However, expression of L-type channels with a surprisingly negative activation threshold has been reported in the hippocampus (Avery and Johnston, 1996; Hasreiter et al., 2014), as well as in other brain regions (Regan et al., 1991).

Of course, given the considerable heterogeneity of intracellular pathways activated by OtRs, we cannot exclude that also other ionic conductances modulated by TGOT could contribute to the depolarizing response in CA1 INs. For example, in a previous work we have shown that in olfactory neuronal cells the activation of OtRs could lead to the inhibition of inward rectifier potassium channels, following activation of PLC through pertussis toxin-resistant G protein (Gravati et al., 2010).

### Effect of TGOT on the Membrane Potential of CA1 Pyramidal Neurons

In contrast to what observed in CA1 INs, at spike threshold most of the PYRs were hyperpolarized by TGOT administration and their firing rate was significantly decreased. Our results suggested an indirect action of TGOT on PYRs since this hyperpolarizing response was completely abolished by the blockade of GABA_A_Rs. Furthermore TGOT was not able to modulate the excitatory transmission since we did not observe any significant difference in frequency, amplitude, and kinetics of sEPSCs recorded from PYRs during TGOT administration. This result suggests that in the CA1 region PYRs do not express OtRs. Also, the long duration of the hyperpolarizing response suggests the involvement of extrasynaptic GABA_A_Rs causing a tonic inhibition that is much more prolonged than that mediated by synaptic GABA_A_Rs (Brickley et al., 1996) and a reduction in neuronal firing well documented in many types of neurons (Walker and Kullmann, 2012).

Besides of a lowering in firing frequency of PYRs, TGOT also caused a rightward shift in the firing rate-to-injected current (F-I) relationship, i.e., an increase in the offset but not in the gain of the F-I relationship, consistent with an up-modulation of tonic GABAergic currents, as shown by Pavlov et al. (2009) for hippocampal PYRs.

Finally, it is noteworthy that in CA1 pyramidal neurons tonic Cl^-^-mediated GABA_A_ current is a preferential target of different modulatory systems. In particular, it is suppressed by activation of adenosine A1Rs (Rombo et al., 2016), whereas it is enhanced by activation of cholinergic (Domínguez et al., 2016) and glutamatergic, (Jiang et al., 2015) receptors, and by nitric oxide modulation (Gasulla and Calvo, 2015). These modulatory activities play an important role in cognitive functions and could also be a target in the control of cell excitability both in physiological processes and in anticonvulsant therapy.

## Conclusion

In conclusion, our results demonstrate that OT plays an important role in the hippocampal network, by modulating the inhibitory transmission onto PYRs located in the CA1 *stratum pyramidale*. OtRs, the molecular target of OT, are mainly expressed in stuttering fast-spiking GABAergic INs, that respond to the agonist with a depolarization and an increase in their firing rate, causing the release of a larger amount of neurotransmitter in the extracellular space. This, in turn, will cause an increase in the amplitude and frequency of sIPSCs elicited in PYRs. In addition, spreading of part of neurotransmitter outside the synapse produces an activation of perisynaptic GABA_A_Rs that, according to their peculiar biophysical features, generate slower sIPSCs. When GABA spillover is massive, the neurotransmitter binds also to extrasynaptic GABA_A_Rs leading to an increase of the tonic GABAergic current. The hyperpolarization induced by the tonic GABAergic current is mainly due to: (i) an increase in the inward chloride current through the extrasynaptic GABA_A_Rs and (ii) a persistent reduction in the input resistance. These phenomena all together cause a reduction in cell excitability and therefore in the capability of PYRs to generate action potentials in response to excitatory inputs.

The proposed mechanism suggests that OT, by influencing the activity of hippocampal GABAergic INs, can regulate the operational modes of the downstream PYRs, possibly not only by modulating their inhibition/disinhibition, but also by improving the signal-to-noise ratio, by inducing and maintaining network oscillations or by promoting plasticity, as already proposed by Freund and Buzsáki (1996); Zaninetti and Raggenbass (2000), and Owen et al. (2013). Furthermore, OT could also be therapeutically used to protect hippocampal synaptic plasticity and memory processes during uncontrollable stress (Lee et al., 2015).

## Ethics Statement

All animal care and experimental procedures were conducted in accordance with the Italian Animals Scientific Procedures Act (Italian Ministry for University and Research – Protocol 523/2018 PR) and with the ethical policy under which Frontiers in cellular neuroscience operates. Animals were housed with food and water *ad libitum*, under a 12:12 h light/dark cycle.

## Author Contributions

All work was performed in GB’s and MT’s laboratory, University of Pavia. GB and MT initiated the study, designed experiments, and wrote the manuscript. CM, FT, and PS performed experiments. CM, FT, and MT analyzed the data. All authors have approved the final version of the manuscript and agreed to be accountable for all aspects of the work, ensuring that questions related to the accuracy or integrity of any part of the work are appropriately investigated and resolved. All persons designated as authors qualify for authorship and all those who qualify for authorship are listed.

## Conflict of Interest Statement

The authors declare that the research was conducted in the absence of any commercial or financial relationships that could be construed as a potential conflict of interest.
